# Remote Teaching Due to COVID-19: An Exploration of Its Effectiveness and Issues

**DOI:** 10.3390/ijerph18052672

**Published:** 2021-03-06

**Authors:** Hiromi Kawasaki, Satoko Yamasaki, Yuko Masuoka, Mika Iwasa, Susumu Fukita, Ryota Matsuyama

**Affiliations:** 1 Department of Public and School Health Nursing, Graduate School of Biomedical and Health Sciences, Hiroshima University, 1-2-3, Kasumi, Minami-ku, Hiroshima 734-8553, Japan; morisato@hiroshima-u.ac.jp (S.Y.); d170821@hiroshima-u.ac.jp (Y.M.); m-iwasa@shitennoji.ac.jp (M.I.); fukita1234@hiroshima-u.ac.jp (S.F.); 2 Department of Health Informatics, Graduate School of Biomedical and Health Sciences, Hiroshima University, 1-2-3, Kasumi, Minami-ku, Hiroshima 734-8553, Japan; rmatsuyama@hiroshima-u.ac.jp

**Keywords:** COVID-19, emergency remote teaching, nursing students, face-to-face classes

## Abstract

Universities have quickly shifted to remote learning due to the COVID-19 pandemic. This study compared two versions—emergency remote teaching (ERT) and conventional face-to-face class (FFC)—of a course design based on the instructional design ARCS model for effectiveness and issues. The current study comprised 46 third-year nursing students who attended an FFC course in 2019, and 56 third-year students who took the ERT version in 2020. Students’ self-rated goal attainment and knowledge of genetics scores were compared before and after taking the courses. Scores between the two class types were compared using the Wilcoxon rank sum test. The students’ worksheets were evaluated using keyword frequency and content analyses. Both classes achieved their goals satisfactorily, and this study confirmed that for this course, ERT was as effective as FFC. A comparison of the increase in domain goal attainment scores per student showed that only the psychomotor domain item, “I can fully explain human diversity using genomic information”, was significantly different, as it was significantly higher for ERT (*p* = 0.003). This higher item in the ERT group suggests that ERT can pose a lack of practice caution in acquiring nursing skills.

## 1. Introduction

In March 2020, due to the COVID-19 pandemic, measures were taken worldwide to limit contact between people, including the suspension of classroom teaching [1]. In Japan, the government declared a state of emergency. However, in the Japanese system, preparations are made for the first term of the new school year in March before it begins in April. Since the campus closures coincided with the start of classes, universities were thrown into disarray [2]. To fulfill their social responsibility to reduce the risk of infection among staff and students, universities shifted to online learning [3]. Healthcare-related educational institutions with campus hospitals were concerned not only for the health of their students but also wanted to avoid the risk of infection among patients with weakened immune systems. To reduce these risks, they converted to online learning practically overnight [4].

Previously, although universities were encouraged to offer classes online (i.e., remote teaching), online education was generally considered inferior to the face-to-face form of teaching [5,6]. This was largely due to issues related to cost and the limited availability of computer-related technologies [7]. Even though these technologies have advanced and are widely available, remote teaching has lower student retention rates and provides less feedback to students than classes taught in a classroom, in the face-to-face form. It has also been suggested that some disciplines may not be suited for online learning [8]. It has been argued that student support cannot be provided adequately in an online environment [9]. However, distance education has become very attractive because of certain advantages, such as study program choice and time efficiency [9]. Comparative studies have already been conducted regarding the effectiveness of remote teaching classes versus face-to-face classes, with results indicating that, in a limited number of domains, there is no difference between the two approaches [10,11,12].

According to Al Lily et al., the effectiveness of remote education systems hastily improvised due to COVID-19 is poor, and such crisis-reactive remote education differs from conventional remote education [2]. As a result of the sudden transition to online learning, leaving no time to analyze the impact on educators (instructors), system design (systems managers), or social impact educators and relevant experts were unable to plan for well-designed online instruction that could have made the transition easier for students and faculty [13].

Moreover, remote learning requires students to possess and apply self-regulation skills. Highly self-regulated learners exhibit effective intrinsic motivation and self-efficacy with respect to their learning process by selecting learning content, identifying learning goals, and organizing and controlling their learning process [14]. Previous studies show that, in online learning, individuals who lack motivation and self-regulation skills may take too much time to complete assignments, resulting in late submissions or poor-quality work [15]. In addition, the social distancing required during the COVID-19 crisis made students more susceptible to anxiety due to isolation [16]. To retain online students, even while they are learning at home alone during this crisis, it is particularly important to use a motivational learning approach [17,18,19].

Researchers have focused on instructional design (ID) and used ID in online learning because it characteristically focuses on self-learning [20]. ID was originally developed, not for designing remote teaching classes, but to increase motivation in face-to-face classrooms. ID aims to maximize the effectiveness of an instructional approach for the learner. That is, the objectives are to effectively increase learning, improve the learning environment, and sustain learner motivation. In particular, the attention, relevance, confidence, and satisfaction (ARCS) model focuses on motivation in learning [21]. The ARCS model design is considered particularly suitable for professional education in classes in areas where it is difficult to motivate students, since the ARCS model emphasizes its relevance to the learner’s expertise. We previously investigated the ARCS model in a conventional face-to-face classroom course for radiational education [22,23] and genetic education [24] and confirmed the usefulness of the ARCS model. To cope with emergencies, we hurriedly converted a conventional face-to-face course, developed using the ARCS model, to a remote reaching course. To implement this “emergency remote course,” we reused PowerPoint presentations prepared previously for the conventional face-to-face course by adding recorded explanations to the slides, along with uploading the handouts and worksheets to the online educational system with no changes to the topics or content.

Since the original face-to-face lecture course designed using the ARCS model was fully converted to the emergency remote course, it is naturally expected that the remote course also maintained the advantages of the ARCS-modeled lecture against the abovementioned consideration in Al Lily et al. [2]. Confirming that the emergency remote course converted from the face-to-face course is as effective as the original course will (i) give lecturers hints about the lesson design in normal times and (ii) reduce the burden on lecturers in emergencies. In order to propose these two benefits, it is necessary to evaluate the possible differences in the effectiveness of the two lecture styles. Therefore, this study aimed to verify whether there were any differences between the face-to-face course developed using the ARCS model before the COVID-19 pandemic and the same course offered as an emergency remote teaching course during the pandemic. Adding to this, the present study also aimed to clarify the educational challenges associated with this transformation of teaching forms.

The term “distance education” has evolved to include several forms of learning, such as online learning, e-learning, and online collaborative learning. In this study, conventional in-person classes taught in a classroom were referred to as “face-to-face class” (FFC) courses, and the online emergency versions due to COVID-19 were referred to as “emergency remote teaching” (ERT) courses [5].

## 2. Materials and Methods

### 2.1. Design

This study used a case-control design, comparing students who attended conventional FFC courses, as controls, with those who took ERT lectures, as cases. It was hypothesized that there is no difference in the effectiveness of lectures between the FFC developed under the ARCS model and the ERT that copied the contents of the FFC.

### 2.2. Recruitment

The target sample consisted of 62 students taking the course in their third year in 2019 and 59 students in their third year in 2020 in the School of Nursing. Although the two groups started school in different years, they completed the same curriculum. The course on knowledge and perceptions related to human genomics was the same, except that the 2019 course was the FFC version and the 2020 course was the ERT version. All the measures were the same between the course groups and were collected pre/post relative to the class in that year. The students were asked to cooperate in the study after their academic grades had been allotted to prevent them from thinking that cooperating in the research was related to their academic evaluation and grades. The procedure for cooperation was carried out by an assistant professor who did not conduct the class. Only those students who had completed their course submissions and expressed their consent to participate were included in the study. The survey was administered when the students completed the submission after this class, and the data were managed by assistant professors who were not involved in assigning academic grades. Students with missing tests and worksheets were excluded.

### 2.3. Overview of Training

#### 2.3.1. Learning Objectives

The subject matter for this course was designed for students new to human genomics, to learn how to link the genetics topics they had already studied in biology [25] with the genetics of human heredity and its relationship to nursing care. In practice, it is challenging for nurses to make a connection between the genetics that they study in biology and their actual nursing activities [26]. Learning objectives were developed using Bloom et al.’s taxonomy of educational goals [27]; these involve setting attainment goals for the three learning domains, namely, cognitive, affective, and psychomotor. Using the study of basic nursing skills and health counseling, we gave students a clinical case related to human genomics, but provided them with minimal information, much less than what is generally provided in such clinical cases. However, to the extent possible, we included such genetics-related content from the nursing student curriculum that was directly relevant to the counseling case [28]. The students needed to recognize from the little information they had that, to respond to the person being counselled, they would need knowledge and skills related to human genomics; not the genetics of fruit flies and green peas they had studied in biology, but the genetics of human heredity. Students were then shown the attainment goals for the exercise. For the cognitive domain, the goal was “to be able to understand genomic diseases.” For the affective domain, the goal was “to acquire an interest in people’s genetics.” For the psychomotor domain, the goal was “to be able to respond to consultees using basic genetic knowledge based on diversity.” In the lecture, we used the example of diabetes as a genomic disease for multifactorial inheritance in the same way as Down’s syndrome.

Subscale items for the cognitive domain, the ability to understand genomic disease:

- I am familiar with the term “human genomics.”
- I can explain diabetes by referring to hereditary and environmental factors.
- I have had the opportunity to obtain accurate information about genomic diseases.

Subordinate questions for the affective domain, an interest in people’s genetics to deepen thinking:

- I am interested in studying human genetics.
- I want to obtain accurate information on genetic disorders.
- I am interested in news and articles related to human genetics.

Subordinate questions for the psychomotor domain, the ability to address the needs of people by using knowledge about genetics:

- I can fully explain human diversity using genomic information.
- I can proactively study and consider human genetics by myself.
- I can respond to concerns raised by a member of the community by using knowledge of genetics.

#### 2.3.2. Learning Content

In the education of nurses and public health nurses, human genomics is included in the basic curriculum [28]. Attempts have been made to make students more aware of how genetics is a part of their everyday lives [29]. Genetics can be used to predict hyperlipidemia in children and to prevent heart disease [30,31]. It also contributes to the treatment of Alzheimer’s disease [31]. The use of genetic testing for finding risks (for example, hyperlipidemia and cancer, among others) has increased the contribution of genetics to public health [30,31,32]. Public health nurses believe that more knowledge about the inheritance of genetic traits would be useful in health promotion. Since genetic counseling is performed by “genetic counselors” at specialized institutions in Japan, even municipal public health nurses do not feel the need to learn much about the genetics of heredity [26]. It is even more difficult for students to see the relationship between a genetics course and nursing care. To strengthen students’ motivation to study genetics, its relevance to nurses’ job functions needs to be reinforced in class [21]. This course was designed to strengthen the students’ perception of the relevance of genetics to nurses’ daily jobs by using cases of patients feeling anxious regarding a genetic issue and to teach counseling skills. However, we introduced a case in which the students could not prepare in advance to use the “flipped classroom” technique. If the students knew they were going to have a consultation on a particular issue in advance, they would prepare for the encounter by learning the basics beforehand. However, if they did not know in advance about the encounter, they would have to produce a response using whatever knowledge they already had. Immediately after class began, students first worked on knowledge tests and case studies. The genetic knowledge and knowledge tests already learned were regarded as part of self-study. The case study was an unexpected consultation rather than a reserved consultation so that students could recognize that they needed to learn basic knowledge. First, students used information that they already had to consider potential responses and, in the process, recognize what they were missing. In this way, students’ motivation to learn is reinforced as the need to learn is clear [21].

##### Case Study

An older adult woman asks the student for advice:

“My 40-year-old niece is pregnant. She is in her third month. I have heard that older women have a greater chance of having a child with Down’s syndrome. Is that something to worry about?”

#### 2.3.3. Teaching Tools in Order of Use

**Figure 1:**
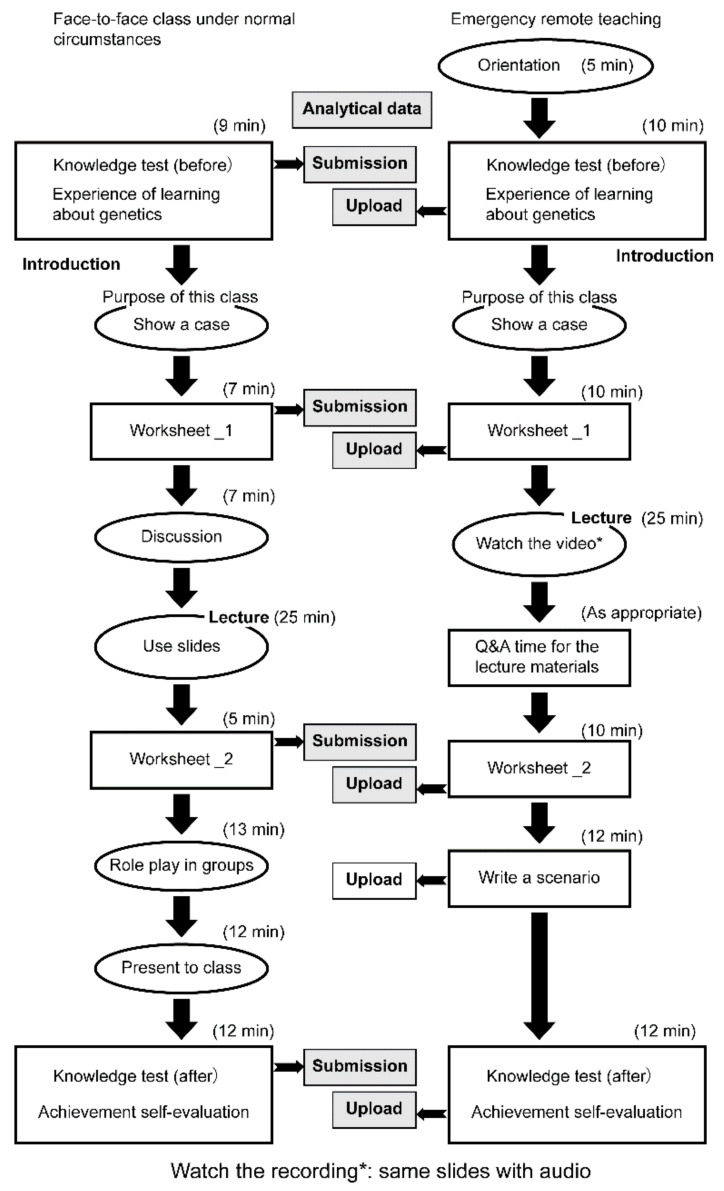
Teaching tools in order of use. * same slide with audio.

The teaching tools used and their order of use are shown in Figure 1.

#### 2.3.4. Lectures

After the students were presented with the request for advice, they were taught the relevant skills and knowledge they would need to respond. They were shown how to assess the person’s situation and intentions and were given accurate information on topics from which to select for their responses. These topics included the role of the human genome, genetic diversity, hereditary disorders, Down’s syndrome, the characteristics of genetic information (invariance, predictability, sharing), and how to understand diversity.

#### 2.3.5. Tools

##### Genetics Knowledge Assessment

The genetics knowledge test included 38 problems consisting of 12 true/false, 12 fill-in-the-blanks, and 14 essay questions. Points were allocated to each problem for a perfect score of 100. The test included fill-in-the-blank questions about the names of multifactorial inheritance disorders and types of Down’s syndromes and their explanations, true/false questions about single nucleotide polymorphisms, and essay questions about characteristics specific to genetic information.

##### Self-Assessment Regarding Attainment Goals

A 5-point Likert scale was used to assess the attainment of course goals. The assessment consisted of three items each for cognitive, affective, and psychomotor domains. The maximum score for an item was 5, with items rated 1 = Not at all true of me; 2 = A little true of me; 3 = True of me half the time; 4 = Quite true of me; and 5 = Very true of me. The items consisted of positive statements about oneself, so higher scores indicated greater achievement. As a result, the maximum score for each domain was 15, and the maximum total score for the three domains was 45. The students performed this self-assessment before and after the course.

##### Worksheets

The course used two worksheets to help students write a response to the case. They were similar in content. Worksheet 1 was completed in the first half of the course, and Worksheet 2 was completed after the lectures had been delivered. The effectiveness of the course, especially in the psychomotor domain, was evaluated from the change in learner response between the two.

##### Scenario

In the ERT version of the course, students wrote a scenario that illustrated how they would respond to the person being counselled instead of performing an actual role-play for practice.

##### Course Design Evaluation

J.M. Keller [21] proposed the ARCS model of ID to integrate many different motivational theories. He considered motivation to learn as having four dimensions: attention, relevance, confidence, and satisfaction. The composition of the course and materials was evaluated by the students. To evaluate the course and teaching materials, the students completed an evaluation sheet based on the ARCS model developed by Kogo and Suzuki [33]. Students responded to each of the four dimensions: Attention, how interesting was the lecture; relevance, the content of the lecture had something to do with me; confidence, I gained confidence in human genetic health counseling; and satisfaction, the content of the lecture was ready to use.

### 2.4. Analysis

The sample used for the analysis consisted of the records of the complete responses received from students consenting to participate in the study. Since at this university, there were no major differences in ability between students who were enrolled in different years [22], the work of students from different years of admission could be compared. The submitted work containing omissions was excluded. Most students were 20−21 years old and female. Of the 62 third-year students who took the FFC course in 2019, 46 (74.2%) agreed to participate in the study, as did 56 (94.9%) of the 59 third-year students who took the ERT version in 2020. First, the students’ mean self-rated goal attainment scores and knowledge of genetics test scores obtained before and after the course were compared by class type to confirm performance attainment. Before and after score comparisons, based on class type, were performed using the Wilcoxon signed rank test. Subsequently, we also compared all the before scores by class type since self-rated assessments can differ based on the type of learning [34]. The ERT scores were higher than the FFC scores before the course. To test for significant differences in effectiveness except for the influence of the baseline, we compared the differences between before and after scores by class type. We used the Wilcoxon rank sum test. All tests were run using IBM SPSS Statistics 25.0 (IBM Corp, Armonk, NY, USA) with a significance level of 5%.

The students used Worksheets 1 and 2 with similar content to write down their thoughts about the case and their responses. To assess the effectiveness of the course, we evaluated the change in each learner’s thinking and their responses between the two worksheets using text mining. The worksheets’ qualitative data were loaded into a text mining software application, and morphological analysis was used to extract parts of speech information. According to the perspective that the number of nouns (keywords) expresses the number of concepts [35], we analyzed the number of nouns in each worksheet and how frequently they were mentioned [36]. Multiple nouns with the same or similar meanings were combined into one keyword. In addition, descriptive content was summarized into concept categories based on the semantic content in each phrase to evaluate goal attainment by class type. Objectivity was ensured by having three researchers conduct this task.

For the analysis of the worksheets, we used IBM SPSS Text Analytics for Surveys 4.0 (IBM Corp, Armonk, NY, USA). The worksheets for 2019 and 2020 were compared in the same way.

After completion of the course, the course design was evaluated by the students using the course evaluation sheet based on the ARCS model [21] developed by Kogo and Suzuki [33]. We examined whether the evaluation of motivation by students differs depending on the class method. Scores for the four dimensions (attention, relevance, confidence, and satisfaction) were compared by class type. The comparison of scores for the FFC and ERT versions of the course was performed using the Wilcoxon rank sum test. For the statistical analysis, we used IBM SPSS Statistics 25.0 (IBM Corp, Armonk, NY, USA) with a significance level of 5%.

### 2.5. Ethical Considerations

Our study was approved by the research ethics review committee of Hiroshima University (approval number: E-1776-2). Students who decided to participate provided written informed consent after receiving an explanation of the study’s purpose and methods, the fact that participation was voluntary, and they could withdraw their participation at any time, and how the data would be treated. Data obtained from consenting participants were used for the study’s analysis, but all data were anonymized by substituting an individual identification number for the participant’s name.

## 3. Results

### 3.1. Evaluation of Course Effectiveness

#### 3.1.1. The Cognitive Domain

The genetics knowledge test had a maximum score of 100. The score before the FFC course was 19.09 (SD 7.03) and, immediately after, it was significantly higher at 71.24 (SD 16.84; *p* < 0.001). The mean score before the ERT course was 34.05 (SD 8.81) and, after the course, it was also significantly higher at 91.34 (SD 9.05; *p* < 0.001). The mean difference for the class before and after scores for the FFC version was 52.15 (SD 16.47) and for the ERT version was 57.29 (SD 9.53). The difference between these scores for the two class types was not significant.

The self-rated goal attainment scores for the cognitive domain are shown by class type in Table 1. Out of a total possible score of 15, the mean total scores before and after the FFC course were 7.67 and 11.02. For the ERT course, they were 9.45 and 12.68. Both increases were significant (*p* < 0.001; Table 1). In addition, scores before the ERT course were higher than those for the FFC course (*p* = 0.053–*p* < 0.001; not shown).

The keyword frequency analysis for the worksheets showed an increase in the number and frequency of keywords used. The most frequently used keyword was Down’s syndrome, which increased from a total of 53 occurrences before to 132 after the FFC course. In the ERT course, occurrences of the keyword Down’s syndrome increased from 112 to 143 (Table 2).

To compare the effectiveness of the FFC and ERT versions of the course, we calculated the difference between before and after goal attainment scores by students for the cognitive domain to investigate the difference by class type and the difference between FFC and ERT, which were 3.35 and 3.23, respectively. The two were not significantly different (*p* = 0.801) (Table 3). In the worksheet analysis, more keywords were found related to understanding diversity in the ERT course than in the FFC course (Table 2).

#### 3.1.2. The Affective Domain

For the self-rated goal attainment for the affective domain, out of a total possible score of 15, the mean scores before and after the FFC course were 8.17 and 9.72. For the ERT course, they were 10.80 and 12.54. For both the FFC and ERT courses, the mean total scores as well as the mean scores for the subscale items significantly increased (*p* < 0.001; Table 1). Table 3 shows a comparison of the change in mean scores per student for the two courses. The difference in FFC was 1.54, and the difference in ERT was 1.73, which was not a significant difference (*p* = 0.551).

#### 3.1.3. The Psychomotor Domain

For self-rated goal attainment in the psychomotor domain, the mean total scores before and after the FFC course were 4.97 and 9.00. The scores for the ERT course were 6.91 and 11.36, respectively. Both increased significantly (*p* < 0.001; Table 1). The mean scores for the subscale items for both courses also increased significantly. No significant difference in effectiveness was found between the class types (Table 3): The difference in FFC was 4.02, and the difference in ERT was 4.45 (*p* = 0.213). However, for the subscale item “I can fully explain human diversity using genomic information,” the difference between class types was significant (*p* = 0.003).

Recognition of the role genetics plays in nursing belongs to the psychomotor domain. To what extent this was recognized was assessed in the worksheet analysis by comparing the content analysis results for Worksheet 2 for the two class types (Table 4). The concept categories common to the two extracted counseling-related lesson methods were: “Providing accurate information,” “Empathetic support,” and “Professional counseling from a doctor or midwife.” “The wonder of giving birth to a child” was extracted only on the FFC, and “Supporting diversity-affirmative parenting” was in the ERT.

### 3.2. Course Design Evaluation

The design of the courses was evaluated by students using a scale based on the ARCS model. The mean scores are shown in Table 5. The means for all four dimensions exceeded 3 out of a total possible score of 5. The highest mean scores were for attention, 3.72 for the FFC course and 3.96 for the ERT course. The only significant difference found between the courses was for confidence, where the mean ERT score was 3.38 and the FFC score was 2.89 (*p* = 0.009).

## 4. Discussion

This study aimed to confirm that the effects of urgently modified distance learning are not different from those of regular classes. Regardless of class type, scores in all domains significantly increased in measures taken after the course compared to before the course. The worksheet content also improved. Based on these results, both versions of the course could be judged as effective. In addition, self-assessment scores before the ERT course were higher than those for the FFC course, which supported the finding in a previous study that self-assessments can differ depending on the learning environment for the course [34]. Online courses have fewer time constraints, so students have more time to think and write compared to FFCs, which facilitate more detailed responses [37]. Although the ERT course in this study also had time constraints, they were less stringent than those of the FFC course. This could be why the initial scores for the ERT course were higher than those for the conventional course. To compare the effectiveness of the class types when the initial scores differed, we decided that it was reasonable to use the difference before and after each class.

The activities in this study made it possible for students to learn how to search for this type of information and to appreciate the need for basic knowledge about genetics. The amount of knowledge that nurses generally possess to guide people who lack that knowledge is important. For nurses to continually provide better care, it is important to acquire the habit of collecting information [38,39]. The knowledge of genetics test scores did not differ depending on the course class type. Worksheet analysis showed that Worksheet 2 completed in the latter half of both courses had more Japanese characters than Worksheet 1 completed in the first half. The change in the number of keywords and the increase in the number of characters suggested there had been cognition-related changes in the students. The high correlation between the number of characters and the number of concepts in text mining is an important indicator of conceptual complexity [35]. The keywords used in the second worksheet were longer (i.e., written with more characters), which showed the development of the concept of genetics. These results suggested that the students had acquired sufficient knowledge regarding genetics to meet the goal set for the cognitive domain.

Nursing staff need to acquire basic knowledge of the sciences and clinical reasoning skills, which will enable them to professionally help people in a wide variety of situations [38,40]. Knowledge of the sciences is essential to nursing practice. Moreover, Birks et al. found that nurses prioritized anatomy and other biological sciences as relatively more important for nursing practice than other topics or branches of science [38]. In this study, even though the students had studied genetics in biology, their pre-course scores were low for the question item “I am familiar with the term human genomics.” In a study on the competencies of genetic nursing practice in Japan, Arimori et al. state that general nurses need to be competent in the areas of “living support, psychological support, and identifying clients’ needs,” and genetic nurses need to be competent in the “provision and exchange of appropriate genetic information” [41]. For general nurses (including registered nurses, public health nurses, and school nurses), it could be that the knowledge of genetics learned in biology is unlikely to result in a better understanding of the health-related situations of humans. However, students can make a connection between genetics in biology and a specific case of nursing care by limiting the amount of genetic knowledge they need to what is relevant to a clinical case. The next stage of training to be a genetic nurse leads to specialized learning to develop that knowledge into an understanding of human genomics [42,43].

General nurses refer people to genetic nurses. It is the general nurse who, as part of their everyday work, is responsible for noticing health problems that can be presumed to be genetic in nature and calling people’s attention to them. It is difficult for people to recognize on their own when they need to see a genetic specialist. Furthermore, genetic specialists, such as genetic counselors and genetic nurses (who specialize in genetic disorders), are not available at medical centers that people usually visit [44], and therefore, people need to seek them out specifically. General nurses are required to provide people with the relevant information and refer them to specialists. In this study, two of the counseling-related categories identified from the worksheets were “providing accurate information” and “professional counseling from a doctor or midwife.” These categories were identified for students in the process of learning the general level of nursing regardless of the course they were in. The students recognized that to advise the person in the case study, they needed specialized knowledge and skills that required them to consider genetics in relation to human heredity. Although the knowledge required for the case study was limited, the students learned enough about genetics to attain the cognitive domain goal, which was “To be able to understand genomic diseases.”

Regardless of class type, the attainment scores for the affective domain were the highest of the three domains. The students learned the need to study genetics, recognizing that nurses sometimes provide information about genetics as part of their daily work. The affective domain corresponds to the attention and relevance dimensions in the course design evaluation [33]. No significant differences were found due to differences in class type. This demonstrated that when ID using the ARCS model has been used to increase student interest regarding human genomics in a course, the course can still be effective even when changed from an FFC to an ERT.

The psychomotor domain is about learning skills and putting them into practice. When an FFC approach is used, the plan is for students to learn through discussion with each other and role-playing. By simulating the experience through role-playing, students develop awareness of behaviors that will help them achieve the goal of “being able to address patients’ needs.” In a counseling situation, the student may be asked for advice on an unexpected subject—something the student cannot prepare for—and the question may, at first, not seem important. However, the person could change their behavior because of the student’s response. Case studies give nursing students an opportunity for experiential learning [45], and that experience can be presumed to result in effective learning in the psychomotor domain. Confidence in one’s nursing skills is believed to develop with practice [46]. However, practice is also affected by one’s motivation to learn [47]. Worksheet content analysis showed qualitative category commonalities and differences. The category that was the same in the two teaching methods was the category related to the role of the nurse, where students described their behavior as a nurse. This means that the goal has been achieved. The categories extracted exclusively for FFC were emotional content: The wonder of giving birth to a child. The categories allowed only for ERT were related to goals: Supporting diversity-affirmative parenting. In FFCs, the emotions of the instructor giving the lecture were conveyed, and in the ERT, it is possible that the students were thinking about their expectations. These differences may affect the conversation and motivation for students’ consultation answers.

Since role-playing makes one more conscious of what one is doing, it should be important to integrate role-playing, or the creation of scenarios simulating role-play, in online courses as well. The effects related to role-playing in the psychomotor domain were confirmed quantitatively and qualitatively for the FFC approach. Although there was no role-playing in the ERT course, the scenario simulations were confirmed to be effective given that regardless of class type, students attained the psychomotor domain goal. Looking closely at the psychomotor domain goal attainment scores, in the ERT course, students only created written scenarios describing the process they would use to respond to the case study question rather than role-playing because of technology-related limitations. The mean score for “I can fully explain human diversity using genomic information” was higher in the ERT course than in the FFC course. The mean ERT score was also significantly higher for “I gained confidence in human genetic health counseling,” the question item for the confidence dimension of the ARCS model in the course design evaluation.

This suggests that, although online courses with no actual practice may result in a lack of understanding of how to apply knowledge in practice, such a cause-and-effect relationship would need to be verified through a more detailed study. If we can successfully perform role-playing in online practice, we should be able to investigate if there is such a causal relationship. Since there is a need to evaluate students’ learning of the psychomotor domain, including attitudes, in ERT classes, it will be difficult to perform detailed evaluations of student behaviors that require further exploration.

The completion rates for online courses differ depending on the field of study [8]. Some argue that distance education, whether on- or off-line, is not a long-term alternative to face-to-face education and hands-on skills training [48]. In nursing education, major emphasis is placed on practical skills training, even in conventional courses. Nursing skills practice is checked at on-site practicums and when applying for a job. Therefore, just as in FFCs, when skills are learned through ERT, there will be a need to find other opportunities to evaluate students’ practical skills, such as when they attend an on-site practicum or are applying for a job.

This study has some limitations. The ERT data used for this study were created and collected electronically owing to the development and spread of technologies that make remote learning possible. There is a need to confirm that all nursing students can learn remotely, which includes any limitations due to their personal circumstances. In the ERT version of the course, there was no role-play or discussion because of technology issues. For future ERT courses, an easy-to-use remote learning application that supports live interactions between students and teachers needs to be developed. ERT technology development may save teachers the effort of collecting data and creating a database, making it easier to assess the success of a lesson. There is always a possibility that to prevent infectious disease, people may be prevented from meeting in person. It is necessary to consider a lesson design that can be changed instantly and remotely in preparation for a health crisis. It is important to clarify the possibility of conducting education remotely and its limitations, which this study sought to do. This study was conducted only on nursing students in their third year. Further research is needed to apply the results to other areas of expertise.

## 5. Conclusions

This study explored the differences between the FFC and ERT versions of a course designed using the ARCS model of ID. The course used counseling for concerns about congenital health problems as the subject matter. The study found that the ERT version of the course was equally effective as the FFC version. The results showed that using a motivational ID approach can be effective in remote learning. Even though, due to technical problems, scenario writing had to be substituted for role-play, course objectives were attained through ERT. Given that students’ self-rated confidence scores were significantly higher in the ERT version than in the FFC version of the course, further testing of online skill enhancement in nursing is warranted.

## Figures and Tables

**Table 1:** Changes in mean scores for goal attainment by type of class.

Learning Domain	Item	Type of Class	*n*	Before		After		*Z*	*p*-Value
				Mean	SD	Mean	SD		
Cognitive	I am familiar with the term “human genomics.”	FFC	46	3.13	0.89	4.11	0.80	−4.55	*p* < 0.001
		ERT	56	3.52	0.85	4.52	0.57	−5.60	*p* < 0.001
	I can explain diabetes by referring to hereditary and environmental factors.	FFC	46	2.28	0.83	3.17	0.85	−4.91	*p* < 0.001
		ERT	56	3.05	0.86	3.91	0.84	−5.20	*p* < 0.001
	I have had the opportunity to obtain accurate information about genomic diseases.	FFC	46	2.26	0.90	3.74	0.80	−5.40	*p* < 0.001
		ERT	56	2.87	1.01	4.25	0.72	−5.70	*p* < 0.001
	Total level of attainment (maximum score: 15).	FFC	46	7.67	1.83	11.02	1.93	−5.68	*p* < 0.001
		ERT	56	9.45	1.93	12.68	1.65	−6.35	*p* < 0.001
Affective	I am interested in studying human genetics.	FFC	46	2.70	0.84	3.17	0.88	−3.25	*p* = 0.001
		ERT	56	3.54	0.85	4.13	0.74	−4.29	*p* < 0.001
	I want to obtain accurate information on genetic disorders.	FFC	46	3.04	1.07	3.52	0.91	−2.75	*p* = 0.006
		ERT	56	3.82	1.03	4.46	0.66	−4.43	*p* < 0.001
	I am interested in news and articles related to human genetics.	FFC	46	2.43	0.78	3.02	0.93	−3.70	*p* < 0.001
		ERT	56	3.45	0.73	3.95	0.77	−4.30	*p* < 0.001
	Total level of attainment (maximum score: 15).	FFC	46	8.17	2.03	9.72	2.41	−4.03	*p* < 0.001
		ERT	56	10.80	2.13	12.54	1.85	−5.27	*p* < 0.001
Psychomotor	I can fully explain human diversity using genomic information.	FFC	46	1.52	0.62	2.98	0.88	−5.80	*p* < 0.001
		ERT	56	2.07	0.74	4.02	0.80	−6.40	*p* < 0.001
	I can proactively study and consider human genetics by myself.	FFC	46	2.00	0.70	3.04	0.92	−5.28	*p* < 0.001
		ERT	56	3.09	0.88	3.87	0.79	−5.52	*p* < 0.001
	I can respond to concerns raised by a member of the community by using knowledge of genetics.	FFC	46	1.46	0.55	2.98	0.72	−5.94	*p* < 0.001
		ERT	56	1.75	0.75	3.46	0.85	−6.27	*p* < 0.001
	Total level of attainment (maximum score: 15).	FFC	46	4.97	1.42	9.00	2.09	−5.87	*p* < 0.001
		ERT	56	6.91	1.73	11.36	2.14	−6.47	*p* < 0.001

FFC: Face-to-face class; ERT: Emergency remote teaching. Wilcoxon signed rank test was used for the comparison.

**Table 2:** Comparison of words (English equivalents) and their total incidence in class worksheets by type of class.

Face-to-Face Class				Emergency Remote Teaching			
Worksheet_1 *		Worksheet_2 **		Worksheet_1 *		Worksheet_2 **	
Word	Number of Words	Word	Number of Words	Word	Number of Words	Word	Number of Words
Down’s syndrome	53	Down’s syndrome	132	Down’s syndrome	112	Down’s syndrome	143
Child	48	Child	90	Child	91	Child	85
Niece	33	Niece	40	Niece	47	Niece	42
Advanced age birth	17	Advanced age birth	29	Advanced age birth	37	Advanced age birth	35
Risk	16	Risk	14	Knowledge	22	Counselee	21
Advanced age pregnancy	13	Gene	14	Risk	18	Uniqueness	17
Feeling	8	Knowledge	14	Counselee	18	Gene	15
Knowledge	8	800–1000 people	13	Probability	16	Probability	15
Counselee	7	Percentage	9	Information	15	Knowledge	15
Age 40	5	Cause	9	The person	14	Disease	12
Probability	5	Age	9	Family	13	Advanced age	11
Idea	5	Mother	9	Advanced age	12	Parents	11
Information	5	Uniqueness	8	Age 40	10	Family	10
Mother	4	Probability	7	Feeling	10	Healthcare	9
Advanced age	3	Feeling	7	Disease	8	Idea	9
Disorder	3	Advanced age pregnancy	7	Disorder	7	Symptoms	9
Age 35	2	Disease	7	Genomic disease	6	Disorder	9
Healthcare	2	Information	7	Pregnancy	6	Genomic disease	8
Birth age	2	Counselee	7	Amniocentesis	6	Feeling	8
Midwifery	2	Factor	6	Congenital	5	Information	8
Doctor	2			Age	5	Age	8
Pregnancy	2					Public welfare	8
Age	2					800–1000 people	7
Appearance	2					Cause	7
						Standard	7

Worksheet_1 *: Students filled this before the lecture; Worksheet_2 **: Students filled this after the lecture.

**Table 3:** Differences in effectiveness by type of class (differences between before and after goal attainment scores).

Learning Domain	Item	Type of Class	*n*	Difference Before and After		Z	*p*-Value
				Mean	SD		
Cognitive	I am familiar with the term “human genomics.	FFC	46	0.98	1.13	−0.220	0.826
		ERT	56	1.00	0.85		
	I can explain diabetes by referring to hereditary and environmental factors.	FFC	46	0.89	0.92	−0.018	0.986
		ERT	56	0.86	0.86		
	I have had the opportunity to obtain accurate information about genomic diseases.	FFC	46	1.48	1.05	−0.271	0.786
		ERT	56	1.38	1.05		
	Total level of attainment (maximum score: 15)	FFC	46	3.35	2.12	−0.252	0.801
		ERT	56	3.23	1.84		
Affective	I am interested in studying human genetics.	FFC	46	0.48	0.89	−0.474	0.635
		ERT	56	0.59	0.85		
	I want to obtain accurate information on genetic disorders.	FFC	46	0.48	1.05	−0.752	0.452
		ERT	56	0.64	0.90		
	I am interested in news and articles related to human genetics.	FFC	46	0.59	0.88	−0.775	0.438
		ERT	56	0.50	0.71		
	Total level of attainment (maximum score: 15)	FFC	46	1.54	2.17	−0.596	0.551
		ERT	56	1.73	1.79		
Psychomotor	I can fully explain human diversity using genomic information.	FFC	46	1.46	0.89	−2.944	0.003
		ERT	56	1.95	0.92		
	I can proactively study and consider human genetics by myself.	FFC	46	1.04	0.82	−1.550	0.121
		ERT	56	0.79	0.73		
	I can respond to concerns raised by a member of the community by using knowledge of genetics.	FFC	46	1.52	0.66	−1.128	0.259
		ERT	56	1.71	1.00		
	Total level of attainment (maximum score: 15)	FFC	46	4.02	1.77	−1.244	0.213
		ERT	56	4.45	2.12		

FFC: Face-to-face class; ERT: Emergency remote teaching. Wilcoxon rank sum test was used for the comparison.

**Table 4:** Categories extracted from worksheets.

Face-to-Face Class		Emergency Remote Teaching	
Category (Number)	Codes (Number)	Category (Number)	Codes (Number)
		Supporting diversity-affirmative parenting (30)	Children’s individuality (26)
			Using public healthcare services (4)
Providing accurate information (26)	Providing information (15)	Providing accurate information (12)	Providing information (8)
	Advanced maternal age-related risks (7)		Risks due to advanced maternal age (4)
	Providing information on prenatal testing (4)		
The wonder of giving birth to a child (10)	The uniqueness of a child’s existence (10)		
Empathetic support (3)	The person and their family (3)	Empathetic support (11)	The person and their family (7)
			Numerous anxieties (4)
Professional counseling from a doctor or midwife (3)	Seek professional advice (3)	Professional counseling from a doctor or midwife (3)	Professional counseling (3)

**Table 5:** Course design evaluation by class type.

	Item	Type of Class	*n*	Mean SD		Z	*p*-Value
**Instructional Design** **ARCS Model**	Attention (The lecture was interesting.)	FFC	46	3.72	0.91	−1.426	0.154
		ERT	56	3.96	0.97		
	Relevance (The content of the lecture had something for me.)	FFC	46	3.61	1.00	−1.559	0.119
		ERT	56	3.91	0.92		
	Confidence (I gained confidence in human genetic health counseling.)	FFC	46	2.89	0.90	−2.613	0.009
		ERT	56	3.38	0.91		
	Satisfaction (The content of the lecture is ready to use.)	FFC	46	3.37	1.02	−1.788	0.074
		ERT	56	3.73	0.92		

FFC: Face-to-face class, ERT: Emergency remote teaching. Wilcoxon rank sum test was used for the comparison.

## Data Availability

The datasets generated and/or analyzed during the current study are not publicly available due to the need to maintain anonymity of participants and the confidentiality of the data. However, the datasets are available from the corresponding author on reasonable request.
